# Force Control and Motor Unit Firing Behavior Following Mental Fatigue in Young Female and Male Adults

**DOI:** 10.3389/fnint.2020.00015

**Published:** 2020-03-31

**Authors:** Katie L. Kowalski, Christie Anita D.

**Affiliations:** School of Kinesiology, University of Western Ontario, London, ON, Canada

**Keywords:** mental fatigue, motor unit firing rate, force steadiness, force control, neuromuscular control

## Abstract

**Purpose**: The neuromuscular mechanisms leading to impaired motor performance in the presence of mental fatigue remain unclear. It is also unknown if mental fatigue differentially impacts motor performance in males and females. The purpose of this study was to assess the impact of mental fatigue on force production and motor unit (MU) firing behavior in males and females.

**Methods**: Nineteen participants performed 10-s isometric dorsiflexion (DF) contractions at 20 and 50% maximum voluntary contraction (MVC) before, during, and after completing 22 min of the psychomotor vigilance task (PVT), to induce mental fatigue. The DF force and indwelling MU firing behavior of the tibialis anterior (TA) was measured before and immediately following the PVT and within the first and final minutes of the PVT.

**Results**: Force steadiness and motor unit firing rate (MUFR) variability did not change during or following the PVT at either contraction intensity (*p* ≥ 0.16). Overall, females had more variability than males in MUFR during the 20% MVCs (15.98 ± 2.19 vs. 13.64 ± 2.19%, *p* = 0.03), though no sex differences were identified during the 50% MVCs (*p* = 0.20). Mean MUFR decreased following mental fatigue in both sexes in the 20% MVC condition (14.79 ± 3.20 vs. 12.92 ± 2.53 Hz, *p* = 0.02), but only in males during the 50% MVC condition (18.65 ± 5.21 vs. 15.03 ± 2.60 Hz, *p* = 0.01).

**Conclusions**: These results suggest possible sex and contraction intensity-specific neuromuscular changes in the presence of mental fatigue.

## Introduction

Mental fatigue is a psychophysiological state that occurs during or after prolonged periods of sustained attention or cognitive activity (Boksem and Tops, 2008) and can present subjectively or behaviorally. Subjectively, mental fatigue is characterized by increased ratings of fatigue (Lim et al., 2010; Hopstaken et al., 2016) or perceived exertion (Marcora et al., 2009; Van Cutsem et al., 2017). Behavioral manifestations of mental fatigue include reductions in task vigilance (Lim and Dinges, 2008) and attention (Boksem et al., 2005; Hopstaken et al., 2016), slowing of reaction time (van der Linden et al., 2003; Lim et al., 2010) and decrements in exercise performance and manual dexterity (Marcora et al., 2009; Duncan et al., 2015). However, the neuromuscular mechanisms contributing to impaired motor performance in the presence of mental fatigue remain unclear.

With increases in time on task and mental fatigue, there are alterations in blood flow (Lim et al., 2010) and activity (Boksem et al., 2005; Lorist et al., 2005) of the anterior cingulate cortex (ACC). Such alterations are likely to impact the activation of the neuromuscular system, given the function of the ACC as a link between cognition and motor control (Paus, 2001). Indeed, changes to electromyographic (EMG) activity have been demonstrated during an isometric handgrip contraction (Bray et al., 2008) and cycling exercise (Pageaux et al., 2015) following a mentally fatiguing task, suggesting mental fatigue leads to changes in muscle activation strategies. While surface EMG measures following mental fatigue give insight into global muscle activity, it has not yet been determined how mental fatigue impacts motor unit (MU) firing behavior. Such information will provide better insights into changes in the neural control properties with mental fatigue, through assessment of MU firing behaviors that cannot be inferred from standard surface EMG.

Further insights about the mechanisms of mental fatigue may be gained from neuromuscular changes during the concurrent performance of a motor and cognitive task as this scenario leads to similar challenges to attentional resources (Leone et al., 2017) and decrements in motor performance (Mehta and Agnew, 2012) as a mentally fatiguing task. The coefficient of variation (CV) of force during a submaximal isometric contraction increases during dual-task conditions, compared with single-task (Lorist et al., 2002; Pereira et al., 2015), suggesting greater oscillations in the common synaptic input to the motor neuron pool (Farina and Negro, 2015) in the presence of a cognitive task. While studies indicate performing concurrent cognitive and motor tasks leads to changes in force control and motor performance, these results are limited to short-duration cognitive tasks and have not been extended to the condition of mental fatigue.

Additionally, during isometric contractions, the CV of force is higher in females than males in both upper and lower limbs (Jakobi et al., 2018). Relevant factors likely contributing to this sex-specific response include muscle strength and agonist-antagonist activity (Jakobi et al., 2018). When a cognitive task is performed concurrently during an elbow flexion isometric contraction, females have a greater increase in the CV of force and demonstrate greater co-activation than males (Pereira et al., 2015), though this finding is less consistent in the ankle dorsiflexors (Vanden Noven et al., 2014). Females also report higher levels of mental fatigue than males (Engberg et al., 2017), perhaps females may demonstrate greater mental fatigue-related declines motor performance than males. Previous studies examining the impact of mental fatigue on subsequent motor performance have not addressed sex-specific responses (Pageaux et al., 2013, 2015). Thus, it is unknown whether mental fatigue differentially impacts motor performance in males and females.

The purpose of this study was to determine if mental fatigue leads to alterations in neuromuscular function in healthy, young males and females. Specifically, we tested whether a mentally fatiguing task that requires sustained attention impacts force production of the dorsiflexors and MU firing behavior of the tibialis anterior (TA), a key muscle involved in the control of balance and walking. We also sought to determine sex-specific neuromuscular responses to mental fatigue. We hypothesized that following a mentally fatiguing task, there would be greater variability in force production and MU firing behavior compared to baseline measurements and that variability would be greater in females than males.

## Materials and Methods

### Participants

Nineteen participants (10 females, 9 males, 23.4 ± 4.4 years old) were recruited from the local university community and did not report illness associated with fatigue, use of medications which alter cognitive or neuromuscular function, a history of cognitive deficiencies including difficulty concentrating, or musculoskeletal or neurologic impairments. All participants refrained from exercise, alcohol and central nervous system stimulant and depressant pharmacological agents within 12 h of participating in this study, and had normal, or corrected to normal, vision. Each participant provided written informed consent following the procedures approved by the Human Subjects Review Board at the University of Oregon and following the standards set by the Declaration of Helsinki.

### Experimental Protocol

Before the start of the experiment, participants completed the Pittsburgh Sleep Quality Index (PSQI), the Multidimensional Fatigue Inventory (MFI) and rated their subjective feeling of tiredness on a 10-point Likert scale (1 = not tired at all, 10 = very tired). Following the mentally fatiguing task, subjects reported their feeling of tiredness on the same Likert scale.

**Figure 1:**
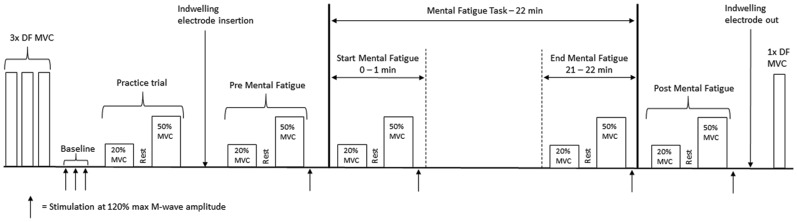
Experimental protocol.

During the single testing session, we obtained measures of dorsiflexion (DF) strength and force variability, surface EMG and indwelling MU firing patterns of the TA before, during and after a sustained attention task that induces mental fatigue. DF muscle contractile properties were also assessed at these time points to ensure no muscular fatigue occurred as a result of the protocol. The experimental protocol is outlined in Figure 1. Baseline assessments of DF maximal voluntary contraction (MVC) force, maximal TA electrical response (M-wave) and DF muscle twitch characteristics were attained before beginning the experimental protocol. Details of these measures are provided below. Following the baseline assessments, participants performed an isometric DF contraction at 20% MVC for 10 s, rested for 6 s, then contracted at 50% MVC for 10 s. Upon relaxation, a single electrical stimulus was applied to the deep peroneal nerve to measure M-wave and muscle twitch characteristics. This series of contractions was carried out before and after completing the sustained attention task as a single-task, and within the first and final minutes as a dual-task. Following the final series of contractions, participants performed a single 4–5 s MVC to ensure no skeletal muscle fatigue had occurred.

### Questionnaires

As sleep quality can impact cognitive and motor functions, the PSQI self-report questionnaire was used to assess overall sleep quality. The PSQI consists of nine self-reported questions about sleeping patterns within the last month. A total score of 5 or greater is indicative of a “poor” sleeper (Buysse et al., 1988). The MFI was used to assess general fatigue. The MFI is a self-report questionnaire with 20 items related to how participants have been feeling “lately” and higher scores indicate greater fatigue (Smets et al., 1995).

### Sustained Attention Task

To induce mental fatigue, participants performed the Psychomotor Vigilance Task (PVT). The PVT is a sustained attention task where participants are instructed to respond to visual stimuli that occur randomly at short intervals (2–10 s) on a computer screen, by clicking a computer mouse as rapidly and accurately as possible (Dinges and Powell, 1985). The PVT is an objective, valid measure for assessing behavioral alertness and vigilant attention (Basner and Dinges, 2011). When performed for 20 min or more, a time-on-task effect is observed with a slowing of reaction time and/or decreases in task accuracy, indicating the presence of mental fatigue (Lim et al., 2010). Lapses in reaction time (RT >500 ms) during this task are associated with subjective measures of physical fatigue and a decline in energy (Lee et al., 2010; Lim et al., 2010).

A computer monitor placed 2 m from the participants was used to display the PVT. A red number appeared on a black screen and began counting up. Participants were instructed to left-click a wireless mouse they held in their dominant hand as quickly as possible when the number appeared. When the button was pressed, the number counter stopped, briefly displayed the participant’s reaction time, then the screen returned to black before the next stimulus was presented.

Participants performed the PVT for 22 min with the first and last minute as a dual-task of responding to the PVT and performing the series of 20 and 50% MVCs. The first and final minutes of the PVT, when contractions and nerve stimulation were occurring, were not included in the assessment of PVT performance. Lapses (>500 ms response time) were counted and reaction time was calculated as an average of reaction times during minutes 1–6 (Start-PVT) and minutes 16–21 (End-PVT) of the PVT (Figure 1). False starts and anticipations (reaction time <100 ms) were excluded from the reaction time averages.

### Force

Participants were placed in a semi-reclined position on a table with their right foot strapped to a custom-built apparatus designed to measure DF force. Because of a recent right ankle sprain, we used the left foot for one subject. The ankle was set at 20° of plantar flexion with the knee supported in a slightly bent position. An inflexible strap was placed across the dorsum of the foot to ensure contractions were isometric. The custom-built dynamometer was equipped with a load cell (SSM-AJ-250; Interface, Scottsdale, AZ, USA) from which the force signal was amplified (PM-1000; DataQ Instruments, Akron, OH, USA) and sampled at 25.6 kHz using a 16-bit A/D converter (NI USB-6251; National Instruments, Austin, TX, USA). Real-time feedback of force production was displayed to participants using DasyLab software (Data Acquisition System Laboratory, DasyTec, USA, Inc., Amherst, NH, USA).

At baseline, participants performed three MVCs each lasting 4–5 s with 1–2 min of rest between contractions. Additional trials were performed if the peak force varied by greater than 10%. The trial with the highest peak force was used as 100% MVC. Target lines were then displayed at 20 and 50% MVC on a computer monitor 2 m away. Participants performed 10 s contractions at each intensity, separated by 6 s, before the start of the PVT (Pre-PVT), during the first (Start-PVT) and final (End-PVT) minutes of the PVT, and immediately following PVT (Post-PVT; Figure 1).

Using a custom-written MatLab program (Mathworks Inc., Natick, MA, USA) the force signal was down-sampled to 1,000 Hz then measures of mean force and force steadiness were calculated over a 5 s window, avoiding the ramp-up and ramp-down portions of the contraction, which were performed at a self-selected rate. Force steadiness during the contractions at 20% and 50% MVC was quantified as the CV of mean force.

### Intramuscular EMG

MU activity was recorded by inserting a four-wire needle electrode into the mid-belly of the TA with the position of the needle adjusted to maximize subject comfort and quality of the signal. This electrode provides three channels of MU recordings and details of its configuration have been reported previously (Kamen et al., 1995). Once the needle was inserted, participants practiced performing isometric contractions at 20 and 50% MVC. The indwelling EMG signal was amplified and bandpass filtered between 1 kHz–10 kHz (P511; Grass Technologies, Warwick, RI, USA) then sampled at 25.6 kHz with a 16-bit A/D converter and DasyLab software. EMGLab software (McGill et al., 2005) was used for multi-channel decomposition of the signal into single MU action potential trains. Files were manually inspected for accuracy of MU identification and firing patterns with modifications made as necessary. A custom-written MatLab program was used to calculate the mean firing rate, and CV of the interspike interval (ISI), excluding doublets (<10 ms) and long ISIs (>200 ms), avoiding the ramp-up and ramp-down portions of the contraction. MUs with fewer than 10 ISIs were excluded from the analysis. Although some MUs may have been identified in multiple contractions, MUs were not tracked across contractions.

### Surface EMG

A wireless surface EMG electrode (Trigno Wireless EMG; Delsys Inc., Natick, MA, USA) was attached over the distal muscle belly of the TA, aligned along the assumed muscle fiber line. The EMG signal was amplified by 909, band-pass filtered at 20–450 Hz and sampled at 25.6 kHz with a 16-bit A/D converter and DasyLab software. A custom-written MatLab program was used to down-sample the sEMG signal to 1,024 Hz and measure M-wave peak-to-peak amplitude and root mean square (RMS) amplitude. A point before and after the M-wave response was manually selected, and the maximum and minimum values within the defined window were determined automatically by the MatLab program to calculate the peak-to-peak amplitude. Mean RMS was calculated over a 5 s window, avoiding the ramp-up and ramp-down portions of the contraction, and expressed as a percent of maximum RMS during MVC trials.

### Electrical Nerve Stimulation

With the participant at rest, the maximal electrical response of the TA was determined using a stimulating electrode secured over the deep peroneal nerve. A single, brief (200 μs) rectangular waveform stimulus was applied to the nerve (DS7A; Digitimer, Limited, Letchworth Garden City, UK) at increasing stimulus intensities until the greatest M-wave peak-to-peak amplitude was achieved. The stimulus intensity was set to 120% of this value to ensure maximal activation. Three stimuli were delivered before the experimental protocol and averaged to determine baseline M-wave amplitude. Muscle contractile properties were determined from the force response to these stimuli. A single stimulus was delivered following each 50% MVC contraction before, during and after the PVT (Figure 1). Twitch force characteristics were calculated using a custom-written MatLab program. Peak force was identified for each twitch and used to normalize each twitch response before calculating the time to peak force and one-half relaxation time. For peak force, the onset of the twitch was manually identified, then the peak of the force trace was automatically detected and the time between the two was calculated. For one-half relaxation time, the time it took for the force to relax from peak to one-half of the peak was automatically determined.

### Statistical Analysis

Shapiro–Wilk tests were used to assess the normality of all outcome measures. Mauchly’s test of sphericity was performed for all variables examined with repeated measures ANOVA. Where the assumption of sphericity was violated, Greenhouse-Geisser corrections were made.

A two-way (sex, time) repeated measures ANOVA was used to evaluate PVT outcomes (reaction time, lapses, false starts, ratings of fatigue); MVC; muscle twitch characteristics; M-wave amplitude; mean force, EMG RMS and motor unit firing rate (MUFR); and the variability in force and MU ISI at each contraction intensity. When necessary, *post hoc* pairwise comparisons were performed using Bonferroni corrections. Independent samples *t*-tests were used to evaluate sex differences in participant characteristics, including age, height, weight, MFI and PSQI scores. Pearson correlation coefficients *(r)* were used to determine associations between changes in PVT outcomes (reaction time, lapses and fatigue rating), mean MUFR and CV of ISI and force. Effect sizes were calculated to determine the magnitude of differences in MVC and M-wave, and are reported as Hedge’s *g* to account for bias in small sample sizes (Lakens, 2013). Interpretation of the Hedge’s *g* effect size uses the same criteria as Cohen’s *d* to determine the magnitude of the effect size (Cohen, 1988). All statistical analyses were performed using SPSS (Version 24; IBM SPSS Statistics, Armonk, NY, USA). Significance was set at *p* ≤ 0.05 and all data are presented as mean ± SD.

## Results

### Participant Characteristics

Participant characteristics are presented in Table 1. Males and females differed only in their height (*p* < 0.001) and weight (*p* = 0.002) with males being taller and heavier than females. No sex differences were identified for age (*p* = 0.78), MVC (*p* = 0.17), BMI (*p* = 0.22), MFI (*p* = 0.75) or PSQI (*p* = 0.57).

**Table 1:** Participant characteristics.

	Females	Males
Age (years)	23.7 ± 4.1	23.1 ± 4.9
Height* (cm)	165.4 ± 54.2	181.5 ± 58.4
Weight* (kg)	63.0 ± 11.7	82.3 ± 11.3
MVC (N)	189.4 ± 39.1	215.0 ± 65.0
BMI (kg/m^2^)	22.9 ± 3.2	25.1 ± 4.0
MFI	59.9 ± 4.6	59.3 ± 3.0
PSQI	3.7 ± 1.5	4.3 ± 2.9

*Indicates significant difference between sexes. Data are presented as mean ± SD. MFI, Multidimensional Fatigue Index; PSQI, Pittsburgh Sleep Quality Index; MVC, Maximum voluntary contraction.

### Sustained Attention Task

PVT outcomes are presented in Table 2. Subjective reports of fatigue increased from an average of 3.0 ± 1.2 before the PVT to 5.0 ± 1.8 after the PVT (*p* < 0.001). However, there was no main effect of sex (*p* = 0.12) and no significant interaction between sex and time (*p* = 0.88). Reaction time significantly lengthened by 14% from the start of the PVT (276.1 ± 31.5 ms) to the end of the PVT (314.2 ± 37.7 ms; *p* < 0.001). There was no main effect of sex (*p* = 0.81) nor an interaction between sex and time (*p* = 0.19). The number of lapses increased from the beginning (0.8 ± 1.1 lapses/person) to the end of the PVT task (1.9 ± 2.5 lapses/person; *p* = 0.01). However, there was no significant difference between sexes (*p* = 0.62) and no significant interaction between sex and time (*p* = 0.60).

**Table 2:** Psychomotor Vigilance Task outcomes.

	Start-PVT	End-PVT
Self-reported fatigue^†^		
Female	3.5 ± 1.2	5.4 ± 1.7
Male	2.4 ± 1.0	4.4 ± 2.0
Reaction time^†^ (ms)		
Female	281.3 ± 32.1	312.6 ± 37.3
Male	270.4 ± 31.8	315.9 ± 40.4
Lapses^†^ (# per person)		
Female	7 (0.7 ± 1.0)	16 (1.6 ± 1.6)
Male	8 (0.9 ± 1.4)	20 (2.2 ± 3.2)
False starts (# per person)		
Female	10 (1.0 ± 1.3)	6 (0.6 ± 0.8)
Male	9 (1.0 ± 1.0)	12 (1.3 ± 1.4)

*^†^Indicates significant main effect of time. Data are presented as mean ± SD*.

### Force

There was no main effect of sex for MVC force (*p* = 0.17), nor a significant interaction between sex and time (*p* = 0.14). MVC did change over time (*p* = 0.001) as participants had a 7.9% higher MVC Post-PVT (217.4 ± 47.2 N) than Pre-PVT (201.5 ± 53.1 N). However, the effect size for this difference was small (*g* = 0.3).

**Figure 2:**
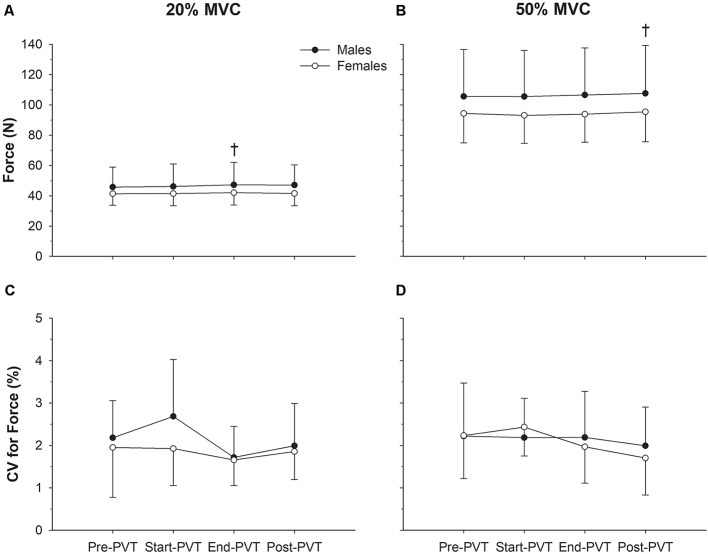
Force characteristics. Mean force in males (closed circles) and females (open circles) across time in the 20% maximum voluntary contraction (MVC; **A**) and 50% MVC **(B)** condition. Force steadiness quantified as the coefficient of variation (CV, %) in males and females across time in the 20% MVC **(C)** and 50% MVC **(D)** condition. ^†^Significantly different than Pre-psychomotor vigilance task (PVT) in panel **(A)** and Start-PVT and End-PVT in panel **(B)**.

Mean force values across time and contraction intensity are presented in Figure 2. In the 20% MVC condition, there was a significant main effect of time (*p* = 0.05). *Post hoc* analysis indicates a trend for End-PVT force (44.5 ± 11.7 N) to be greater than Pre-PVT force (43.5 ± 10.6 N; *p* = 0.06) by 2.5%. There was no significant main effect of sex (*p* = 0.35) or interaction between sex and time for mean force in the 20% MVC condition (*p* = 0.52).

In the 50% MVC condition there was a main effect of time for mean force (*p* = 0.004) with Post-PVT force (101.2 ± 26.1 N) being higher than Start-PVT (99.0 ± 25.0 N; *p* = 0.009) by 2.2% and End-PVT (99.9 ± 25.4 N; *p* = 0.05) by 1.3%. There was no significant main effect of sex (*p* = 0.31) or interaction between sex and time for mean force in the 50% MVC condition (*p* = 0.62).

CV of force across time and contraction intensity is presented in Figure 2. In the 20% MVC condition, CV of force was not significantly different over time (*p* = 0.16) or between sexes (*p* = 0.29), and there was no significant interaction between sex and time (*p* = 0.57). In the 50% MVC condition, CV of force was also not significantly different over time (*p* = 0.29) or between sexes (*p* = 0.84), and there was no significant interaction between sex and time (*p* = 0.62).

### Intramuscular EMG

One-hundred and eighty-eight MUs were evaluated in the 20% MVC conditions and 198 in the 50% MVC conditions. The total number of MUs identified per contraction are presented in Table 3.

**Table 3:** EMG and muscle twitch characteristics.

	Pre-PVT	Start-PVT	End-PVT	Post-PVT
No. motor units				
20% MVC	58	43	47	40
50% MVC	53	53	52	40
RMS 20% (% MVC)^†^				
Female	35.2 ± 14.3	35.3 ± 14.4	35.2 ± 14.6	33.6 ± 14.8
Male	25.7 ± 7.3	24.7 ± 7.8	25.5 ± 7.7	24.0 ± 7.2
RMS 50% (% MVC)*				
Female	57.4 ± 15.2	58.7 ± 14.8	53.6 ± 12.0	52.9 ± 13.9
Male	45.4 ± 17.6	41.7 ± 13.3	41.3 ± 13.6	40.3 ± 12.6
M_Max_(mV)^†^				
Female	1.7 ± 0.8	1.7 ± 0.8	1.7 ± 0.8	1.7 ± 0.8
Male	2.7 ± 1.4	2.7 ± 1.4	2.8 ± 1.5	2.9 ± 1.5
PT (N)				
Female	15.3 ± 5.1	15.3 ± 5.1	15.3 ± 5.1	15.3 ± 5.1
Male	17.3 ± 5.4	17.3 ± 5.4	17.2 ± 5.4	17.2 ± 5.4
TTP (ms)				
Female	79.7 ± 8.8	80.5 ± 8.6	81.7 ± 9.4	80.9 ± 9.4
Male	77.2 ± 11.1	77.4 ± 10.1	77.1 ± 11.1	77.0 ± 11.0
½RT (ms)				
Female	57.1 ± 13.1	57.1 ± 12.4	56.5 ± 12.6	56.9 ± 12.9
Male	53.7 ± 27.6	53.6 ± 27.7	53.9 ± 26.7	54.0 ± 27.5

^†^indicates significant main effect of time. *Indicates significant main effect of sex. Data are presented as mean ± SD. RMS, root mean square; PT, peak twitch force; TTP, time to peak twitch force; ½RT, one-half relaxation time.

**Figure 3:**
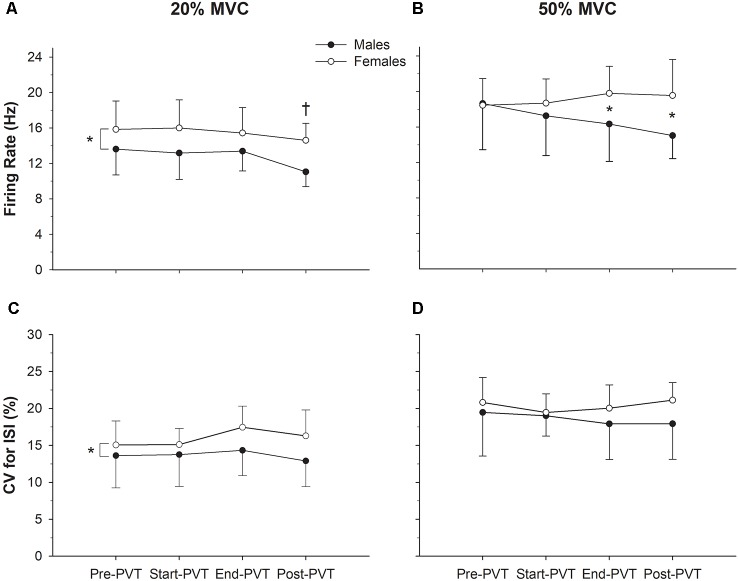
Motor unit (MU) characteristics. Motor unit firing rate (MUFR) in males (closed circles) and females (open circles) across time in the 20% MVC **(A)** and 50% MVC **(B)** condition. Variability of firing rate quantified as the (CV, %) in males and females across time in the 20% MVC **(C)** and 50% MVC **(D)** conditions. *Significant difference between sexes; ^†^significantly different from Pre-PVT and End-PVT.

**Figure 4:**
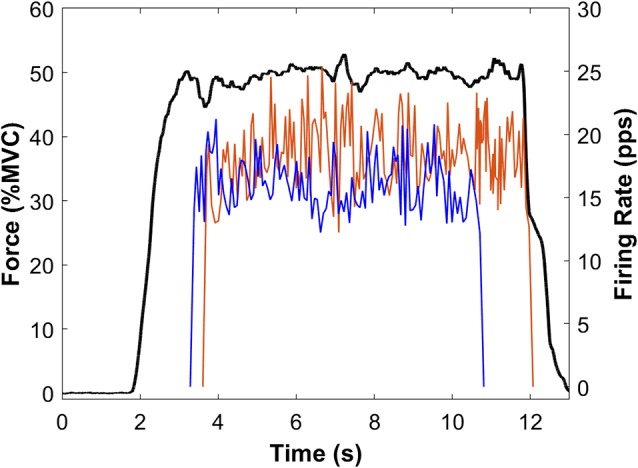
Sample force and MU data from a single participant. Force (black line) is the average of Pre-PVT and Post-PVT trials for this participant. Instantaneous MUFRs are presented for the Pre-PVT (red line) and Post-PVT (blue line) trials. For each trial (Pre-PVT and Post-PVT), the instantaneous firing rates are shown as the average of two MUs.

Mean MUFR across time and contraction intensity are presented in Figure 3, and in Figure 4 exemplary data from a single participant are shown. In the 20% MVC condition, there was a main effect of time (*p* = 0.002) such that mean MUFR was lower Post-PVT (12.9 ± 2.5 Hz) compared to Pre-PVT (14.8 ± 3.2 Hz, *p* = 0.02) and End-PVT (14.5 ± 2.7 Hz, *p* = 0.04). There was also a significant main effect of sex (*p* = 0.02) with a higher MUFR in females (15.5 ± 2.8 Hz) than males (12.8 ± 2.6 Hz). There was no significant interaction between sex and time for MUFR in the 20% MVC condition (*p* = 0.49).

At 50% MVC, there was no significant main effect of time (*p* = 0.54) or sex (*p* = 0.11) for mean MUFR. There was a significant sex by time interaction in the 50% MVC condition (*p* = 0.04). Males and females had similar mean MUFRs during the Pre- (*p* = 0.92) and Start-PVT (*p* = 0.41) trials. However, males had a lower MUFR than females during End-PVT (*p* = 0.05) and Post-PVT (*p* = 0.01).

The CV of MU ISI across time and contraction intensity is presented in Figure 3. In the 20% MVC condition, there was no significant change in CV ISI over time (*p* = 0.37), however, there was a significant main effect of sex with females having a higher CV ISI compared with males (*p* = 0.03). There was no significant interaction between sex and time (*p* = 0.63) in the 20% MVC condition.

In the 50% MVC condition, there was no significant main effect of time (*p* = 0.65) or sex (*p* = 0.20) for CV of MU ISI. There was also no significant time by sex interaction (*p* = 0.53) in the 50% MVC condition.

### Surface EMG

Mean EMG RMS values across time and contraction intensity are presented in Table 3. In the 20% MVC condition there was a significant main effect of time (*p* = 0.02) with *post hoc* analysis indicating a trend for Post-PVT (29.0 ± 12.5% MVC) being lower than End-PVT (30.6 ± 12.6% MVC, *p* = 0.06). There was no main effect of sex (*p* = 0.08) nor an interaction between sex and time (*p* = 0.75). In the 50% MVC condition, the main effect of time was trending towards significance (*p* = 0.06) and the main effect of sex was significant with females having a higher EMG RMS than males (*p* = 0.04). There was no significant interaction between time and sex (*p* = 0.46).

### M-wave and Muscle Twitch Properties

M-wave amplitude and muscle twitch characteristics are presented in Table 3 for a subset of participants (7F, 7M) as a stable M-wave could not be identified in some participants, due to technical difficulties. There was a significant main effect of time for M-wave peak-to-peak amplitude (*p* = 0.01), however *post hoc* analysis demonstrated only a trend towards Post-PVT amplitude being larger than Start-PVT amplitude (*p* = 0.06). There was no significant main effect of sex (*p* = 0.10) or interaction between sex and time (*p* = 0.30) for M-wave amplitude. Muscle twitch characteristics did not vary by time, including PT (*p* = 0.07), TTP (*p* = 0.46) or $\frac{1}{2}$RT (*p* = 0.85). There was no significant main effect of sex on PT (*p* = 0.50), TTP (*p* = 0.52) and $\frac{1}{2}$RT (*p* = 0.79). As well, there was no significant interaction between sex and time for PT (*p* = 0.81), TTP (*p* = 0.30) or $\frac{1}{2}$RT (*p* = 0.56).

### Correlations

There were no significant correlations between the change in subjective reports of fatigue, number of lapses or reaction time and change in the CV of ISI, CV of force or mean MUFR for 20 and 50% MVC conditions (*p* ≥ 0.06).

## Discussion

The purpose of this study was to determine the impact of a sustained attention task on force control and MU firing behavior in young adults and identify sex-specific responses. The results suggest that a sustained attention task-induced mental fatigue, as indicated by increased subjective reports of fatigue and a slowing of reaction time on the PVT, but did not lead to changes in force steadiness or variability of MUFR. However, after the sustained attention task, mean MUFR decreased in both sexes at 20% MVC and only in males at 50% MVC, despite a slight increase in mean force in both sexes.

### Sustained Attention Task

Time on task and sustained attention have previously been shown to induce mental fatigue as indicated by increased feelings of fatigue (Lim et al., 2010; Hopstaken et al., 2016) with a slowing of reaction time (van der Linden et al., 2003; Lim et al., 2010). In the present study, following 22 min of performing the PVT, participants reported a 19.5% increase in subjective fatigue on a 10-point Likert scale. This increase is slightly greater than that reported by Pageaux et al. (2015) who demonstrated an increase in subjective fatigue of approximately 14% following 30 min of a modified incongruent Stroop task. In the current study, at the end of the PVT, reaction time was slowed by 14% and the number of lapses in reaction time more than doubled, which is consistent with others who have used 20 min of the PVT to induce mental fatigue (Lim et al., 2010). Taken together, these findings suggest we induced a level of mental fatigue that was similar to previous reports, through 22 min of the PVT as a sustained attention task.

### Force

Despite males being 12% stronger than females, the DF MVC force was not statistically different between sexes. There are reports of males having significantly greater TA strength than females (Belanger et al., 1983), however, the experimental set up could influence MVC strength due to sex differences in the TA length-tension relationship. The optimal joint position for TA MVC force is 25° of plantarflexion for females and 10° of plantarflexion in males (Belanger et al., 1983). While others who have demonstrated significant sex differences in TA MVC force have used an experimental set up at 0° of plantarflexion (Vanden Noven et al., 2014; Yoon et al., 2014), we used 20° of plantar flexion, which could have biased females towards being closer to an optimal position for MVC strength than males.

We also saw a small, but significant, increase in MVC Post-PVT compared with Pre-PVT. The 8% increase in MVC force was within the margin of error (10%) we used during baseline assessment to establish participant’s MVC. The effect size for the difference in MVC force was small (*g* = 0.31), and therefore it is unlikely that this slight increase in MVC impacted the results of this study. No decline in MVC, combined with a lack of change in contractile properties, suggests that our protocol did not induce peripheral fatigue in the DF muscles and thus any changes in motor output are not a result of peripheral fatigue. These results provide further evidence that mental fatigue does not impair maximal force production (Pageaux et al., 2015; Martin et al., 2015).

Despite setting 20 and 50% MVC target lines, the mean absolute force was significantly different over time. In the 20% MVC condition, the mean force increased by 2.5% during End-PVT compared to Pre-PVT. In the 50% MVC condition, the mean force increased during Post-PVT compared to Start-PVT by 2.2% and End-PVT by 1.3%. While these changes in mean force were small, it is interesting to note that all significant changes in mean force occurred at the end or after the mentally fatiguing task. Perhaps the presence of mental fatigue makes it more challenging to accurately produce force at a given target level. Indeed, others have demonstrated that mental fatigue leads to reductions in more complex movements such as arm pointing tasks (Rozand et al., 2015) and sport-specific tasks such as soccer passing (Smith et al., 2016). Due to its extensive connections with the primary motor cortex and prefrontal cortex, prolonged activation of the ACC during sustained attention and mentally fatiguing tasks is thought to lead to impairments in motor control (Paus, 2001). Twenty minutes of PVT performance leads to an increase in blood flow to the ACC during the PVT, followed by a reduction in blood flow after the PVT (Lim et al., 2010). Prolonged activation of the ACC during our experimental protocol could have contributed to the observed changes in mean force after PVT performance. Additionally, the ACC serves as an error-monitoring system (Rushworth et al., 2004), and thus with the decline in ACC activity after the PVT, possibly our participants had a reduced ability to recognize they were not hitting the target lines and make appropriate corrections. Further investigations involving more complex contractions are warranted to determine the role of mental fatigue in impairing motor control.

Variability in force about a given value is thought to be due to variability in common drive to the motor neuron pool (Negro et al., 2009). In the present study, DF force steadiness did not change as a result of performing a mentally fatiguing task. While this finding is contrary to our hypothesis, it is in agreement with Shortz and Mehta (2017) who demonstrated no change in force steadiness during an intermittent handgrip exercise to voluntary exhaustion after a cognitively fatiguing task in young and older women (Shortz and Mehta, 2017). The lack of identified changes in force steadiness suggests mental fatigue does not lead to changes in the variability in common drive to the motor neuron pool, despite possible alterations to cortical regions responsible for the control of movement.

We also found no difference in force steadiness between the single and dual-task portions of this study. Previous studies have demonstrated that adding a challenging cognitive task while performing an isometric contraction decreases force steadiness in the elbow flexors (Pereira et al., 2015) and first dorsal interosseus muscle (Lorist et al., 2002). Our observed lack of change in force steadiness under dual-task conditions is consistent with other investigations of the dorsiflexor muscle group where force steadiness did not change during dual-task conditions with low to moderate DF contractions in young adults (Vanden Noven et al., 2014). It is also possible the PVT does not provide a sufficient challenge to cognitive resources to lead to declines in force steadiness during the dual-task condition. Nonetheless, our results suggest that the performance of a mentally fatiguing task did not lead to changes in force steadiness for short duration isometric DF contractions during single or dual-task conditions.

Additionally, we did not observe a sex difference for the variability of force production. Previous studies (Brown et al., 2010) have shown that young females have less steady force production than males. However, it was noted that force steadiness had a strong negative correlation with absolute strength, such that the stronger males were steadier than the less-strong females. In the current study, there were no sex differences in DF strength which may explain our observed lack of sex differences in DF force steadiness. Yoon et al. (2014) have also demonstrated no significant difference in DF force steadiness between males and females during a single-task isometric contraction, which was attributed to similar levels of brain activation during the isometric contractions. Our results extend these previous findings to suggest that a mentally fatiguing task does not differentially impact force steadiness in males and females.

### EMG

Mean MUFR was significantly lower Post-PVT compared to Pre- and End-PVT in the 20% MVC condition for both sexes and only in males in the 50% MVC condition. It is an unlikely slowing of MUFR was a result of muscular fatigue as the Post-PVT MVC and contractile properties do not demonstrate muscular fatigue. In the 20% condition, EMG RMS amplitude was also reduced Post-PVT, while there was a trend for EMG RMS to decline at End-PVT compared to Start-PVT in the 50% condition. The reduction in MUFR and EMG RMS amplitude occurred despite a small, but significant, increase in absolute force at both contraction intensities. These results suggest that the presence of mental fatigue altered neural activation strategies, which could be due to prolonged activation of the ACC and its extensive connections with motor planning regions of the brain such as the prefrontal cortex and supplementary motor areas (Paus, 2001; Rushworth et al., 2004; Mostofsky and Simmonds, 2008). Possibly the activation of synergistic muscle groups or changes to co-contraction contributed to the observed changes in surface and indwelling EMG, as has been noted in dual-task paradigms (Pereira et al., 2015). Additional MUs could also have been recruited to maintain force, which would maintain EMG RMS amplitude while MUFR decreased. However, the exact source cannot be determined from the results of this study and future investigation is warranted.

In the current study, females had higher MUFRs than males throughout the 20% MVC condition and during End- and Post-PVT contractions in the 50% MVC condition, with higher EMG RMS throughout the 50% MVC condition than males. Previous research investigating the neuromuscular mechanisms leading to performance decrements following mental fatigue has not addressed sex-specific responses (Pageaux et al., 2013, 2015). However, sex differences have been noted in examinations of neuromuscular function during a concurrent cognitive and motor task. Pereira et al. (2015) demonstrated sex-specific muscle activation patterns, with females using more co-contraction to maintain an isometric contraction than males during a dual-task condition. While the level of co-contraction cannot be determined from the current investigation, our results suggest potential sex differences in the neural control response to mental fatigue and further work is necessary in this area to better understand sex-specific responses to mental fatigue.

Our results also suggest that the performance of a mentally fatiguing task does not impact the variability of MUFR during DF contractions as we found no changes in the variability of MUFR over time in either the 20 or 50% MVC conditions. During a concurrent arithmetic task and wrist extension isometric contraction, Bensoussan et al. (2012) have demonstrated a decline in the variability of MUFR which was accompanied by an increase in MUFR. It is, therefore, possible there are different cognitive and motor responses to a stressful mental arithmetic task and a task designed to induce mental fatigue. However, to our knowledge, the current study is the first to examine MU firing behavior during mental fatigue.

We observed a main effect of sex on the variability in MUFR, such that females had greater variability in their MUFR than males in the 20% MVC condition, though this was not observed in the 50% MVC condition. Similarly, at very low contraction intensities and using high-density surface EMG, Pereira et al. (2019) demonstrated females have greater oscillations in the common synaptic input than males during single- and dual-task conditions. There is a general paucity of data on sex differences in variability of MUFR at different contraction intensities, thus making the intensity-dependent sex difference observed here a novel observation that should be explored further.

### M-Wave and Muscle Twitch Properties

In this study we found that a sustained attention task that induced mental fatigue had no effect on muscle twitch properties as a single or dual-task, indicating our contraction protocol did not induce muscular fatigue. This finding is also consistent with previous investigations in which inducing mental fatigue did not change muscle contractile properties of the knee extensors (Pageaux et al., 2013; Silva-Cavalcante et al., 2018). There was a significant main effect of time for M-wave amplitude, however, given *post hoc* analysis did not demonstrate significant change over time, this difference is likely not significant. No change in M-wave amplitude after a mentally fatiguing task is consistent with the findings of others (Pageaux et al., 2013; Silva-Cavalcante et al., 2018). These results suggest that exercise performance decrements after a mentally fatiguing task are not likely due to impaired contractile properties, and thus more central processes are contributing.

### Limitations

While this study provides further insight into neuromuscular function changes following mental fatigue, there are some limitations. Employing a control day may provide added support to our interpretation of the impact of mental fatigue on neuromuscular properties. However, previous work (Pageaux et al., 2013) has demonstrated that watching a documentary did not change neurophysiological measures, providing the support that the observed changes in our study were indeed due to the effects of mental fatigue.

Although our participants were generally recreationally active, we did not collect physical activity data. Some reports suggest that highly trained athletes are more resistant to the negative motor performance repercussions of mental fatigue (Martin et al., 2016). Future research is therefore warranted to delineate the role of physical activity in modulating the relationship between mental fatigue and neuromuscular function.

## Conclusions

The performance of a mentally fatiguing task did not lead to changes in the variability of force or MUFR. However, surface and indwelling EMG results suggest there are alterations in neural activation strategies, including a decline in MUFR in both sexes during low force contractions, and only in men during moderate-intensity contractions. Our results indicate possible sex and contraction intensity-specific changes in neuromuscular activation patterns in the presence of mental fatigue. Further investigation is required to fully explore neuromuscular function changes in the presence of mental fatigue and determine if these changes contribute to impaired motor performance in the presence of mental fatigue.

## Data Availability Statement

The datasets generated for this study are available on request to the corresponding author.

## Ethics Statement

The studies involving human participants were reviewed and approved by Institutional Review Board, University of Oregon. The patients/participants provided their written informed consent to participate in this study.

## Author Contributions

AC and KK conceived and designed the research, edited and revised the manuscript, and interpreted results of experiments and approved the final version of the manuscript. KK performed experiments, analyzed the data, prepared figures and drafted the manuscript.

## Conflict of Interest

The authors declare that the research was conducted in the absence of any commercial or financial relationships that could be construed as a potential conflict of interest.
